# Preparation and Performance of Radiata-Pine-Derived Polyvinyl Alcohol/Carbon Quantum Dots Fluorescent Films

**DOI:** 10.3390/ma13010067

**Published:** 2019-12-21

**Authors:** Li Xu, Yushu Zhang, Haiqing Pan, Nan Xu, Changtong Mei, Haiyan Mao, Wenqing Zhang, Jiabin Cai, Changyan Xu

**Affiliations:** 1 College of Materials Science and Engineering, Nanjing Forestry University, Nanjing 210037, China; zhangyushu605@163.com (Y.Z.); phq625238728@163.com (H.P.); xunan@njfu.edu.cn (N.X.); mei@njfu.edu.cn (C.M.); maohaiyan@berkeley.edu (H.M.); nldfloor@163.com (J.C.); 2 Jiangsu Co-Innovation Center of Efficient Processing and Utilization of Forest Products, Nanjing Forestry University, Nanjing 210037, China; 3 Jiangsu Province Key Laboratory of Green Biomass-based Fuels and Chemicals, Nanjing 210037, China; 4 Department of Chemical and Biomolecular Engineering, University of California, Berkeley, CA 94720, USA; 5 Jiangsu Chenguang Coating Co., Ltd., Changzhou 213164, China; 6 Jiangsu Province Taizhou Efficient Processing Engineering Technology Research Center for Radiata Pine, Taizhou 214500, China; 13817330966@163.com

**Keywords:** tensile properties, UV barrier, water-resistance

## Abstract

In this study, the low-cost processing residue of Radiata pine (*Pinus radiata D. Don*) was used as the lone carbon source for synthesis of CQDs (Carbon quantum dots) with a QY (The quantum yield of the CQDs) of 1.60%. The CQDs were obtained by the hydrothermal method, and +a PVA-based biofilm was prepared by the fluidized drying method. The effects of CQDs and CNF (cellulose nanofibers) content on the morphology, optical, mechanical, water-resistance, and wettability properties of the PVA/CQDs and PVA/CNF/CQDs films are discussed. The results revealed that, when the excitation wavelength was increased from 340 to 390 nm, the emission peak became slightly red-shifted, which was induced by the condensation between CQDs and PVA. The PVA composite films showed an increase in fluorescence intensity with the addition of the CNF and CQDs to polymers. The chemical structure of prepared films was determined by the FTIR spectroscopy, and no new chemical bonds were formed. In addition, the UV transmittance was inversely proportional to the change of CQDs content, which indicated that CQDs improved the UV barrier properties of the films. Furthermore, embedding CQDs Nano-materials and CNF into the PVA matrix improved the mechanical behavior of the Nano-composite. Tensile modulus and strength at break increased significantly with increasing the concentration of CQDs Nano-materials inside the Nano-composite, which was due to the increased in the density of crosslinking behavior. With the increase of CQDs content (>1 mL), the water absorption and surface contact angle of the prepared films decreased gradually, and the water-resistance and surface wettability of the films were improved. Therefore, PVA/CNF/CQDs bio-nanocomposite films could be used to prepare anti-counterfeiting, high-transparency, and ultraviolet-resistant composites, which have potential applications in ecological packaging materials.

## 1. Introduction

Polyvinyl alcohol (PVA) is a kind of semi-crystalline polymer with a linear structure and strong hydrogen bonds intermolecular. It has been widely used in papermaking [1], textiles [2], coatings [3], adhesives [4], packaging [5], and biomedicine [6] because of its toughness [7], adhesiveness [8], biocompatibility [9], swelling behavior [10], lack of toxicity [11], and sufficient thermal stability; it is also odorless and tasteless. However, PVA is highly hydrophilic and water-soluble [12], and its light transmittance is not suitable for light-barrier packaging [13]. In order to expand the application of PVA, much attention has been paid to fabricating functional PVA composites. The existence of hydrogen-bonding groups in PVA structure and the ability to form hydrogen bonds makes PVA suitable for mixing with other materials to improve its functional properties [14,15]. In previous studies, the prepared silica in situ enhanced PVA/chitosan biodegradation films [16], PVA/tea polyphenol composite films [17], PVA reinforced with cellulose nanocrystals or cellulose nanofibers (CNF) [18], and CNF/PVA-borax hybrid foams [19] had better functional properties than PVA itself, from the perspective of packaging-industry application.

In recent years, carbon materials have been paid much attention for the preparation of functional composites. As a typical carbon material, nanocellulose (CNF) has been successfully used to improve the properties of PVA films because of its nanometer size in diameter, high strength, excellent stiffness, renewability, and high surface area [20,21,22,23]. In addition, the raw materials that can be used to extract CNF are inexhaustible in nature [24]. In particular, PVA contains a lot of hydroxyl, which are accessible for surface modification [23,25]. Carbon quantum dot (CQD) is a new kind of carbon material that emerged in recent years. Since its discovery, CQD has been known as an excellent candidate for diverse applications such as optoelectronics [26], detection of transition metal ions [27], fluorescent inks [28], and cellular imaging [29] due to its strong photoluminescence [30], high photostability [31], good water-solubility [32], low cytotoxicity [33], excellent biocompatibility [34], and environmental friendliness [35]. With persistent efforts, researchers have successfully extracted CQDs from agricultural and forestry waste, including mustard seeds [36], *Actinidia deliciosa* [37], and unripe peach [38]. Recently a few researchers have aimed to make PVA/CQDs composite films. Dong et al. prepared CQDs by hydrothermal reaction. It was added to the PVA solution, and the poly (vinylidene fluoride) film pretreated with an alkaline solution was immersed in the prepared CQD/PVA solution, to coat the surface of the film with a UV-shielding layer, and the composite film could shield the UV light completely [39]. El-Shamy et al. successfully fabricated novel P-type thermoelectric PVA/CQDs nanocomposite films through the solution casting technique. Thermoelectric properties such as electrical conductivity, Seebeck coefficient, and thermal conductivity of the prepared films were studied. These nanocomposites have a promising potential application in thermoelectric devices because they are economical and easy to scale up [40].

Radiata pine is most widely distributed in New Zealand, Australia, and Spain, and it is a major cultivated species in Argentina, Chile, Uruguay, Kenya, and South Africa. Radiata pine wood can be used in construction, wood-based panel, papermaking, furniture, railway sleepers, and other aspects because of its medium density, uniform structure, strong stability, nail holding strength, and good permeability. However, radiata pine is still a new tree species in China. Data from the production line show that about 13% of sawdust is generated when processing 1 m^3^ of logs into sawdust, and about 18% of the shavings and sawdust is produced while making wood structures with 1 m^3^ of sawn timber. Such a large amount of processing residue not only causes great waste of resources, but also pollutes the factory environment and becomes a potential fire hazard. This study provides a new way for efficient utilization of these wastes in extracting carbon quantum dots (CQDs) from them with a simple hydrothermal method [41,42] and preparing PVA/CNF/CQDs fluorescent films. The influence of CQDs load on photoluminescent properties, UV-vis absorption characteristics, tensile property, surface contact angle to distilled water, and water-resistance of PVA/CQDs films and PVA/CNF/CQDs composites were investigated.

## 2. Materials and Methods

### 2.1. Materials

Radiata pine wood (*Pinus radiata* D. Don) processing residue was procured from Jingjiang Guolin Forest Co., Ltd. (Jiangsu, China). After being oven-dried at 105 °C, until its water content was less than 15%, the residue was crushed with a pulverizer (400 Y, Yongkang boao hardware products Co. Ltd., Zhejiang, China) and then passed through a 90-mesh stainless-steel sieve. The resulting wood powder was sealed in a plastic bag, for later use. Coniferous nanocellulose suspension was offered by Zhongshan Nan Fiber New Material Co., Ltd. (Guangdong, China). Its solid content, fiber diameter, and aspect ratio were 2.5 ± 0.5 wt.%, 30 nm, and ≥20, respectively. Both PVA (degree of polymerization, 750 ± 50) and quinine sulfate (99.4%) were purchased from Aladdin Biochemical Co., Ltd. (Shanghai, China). All chemicals were used as received, without further purification.

### 2.2. Synthesis of CQDs from Radiata Pine Processing Waste

**Figure 1:**
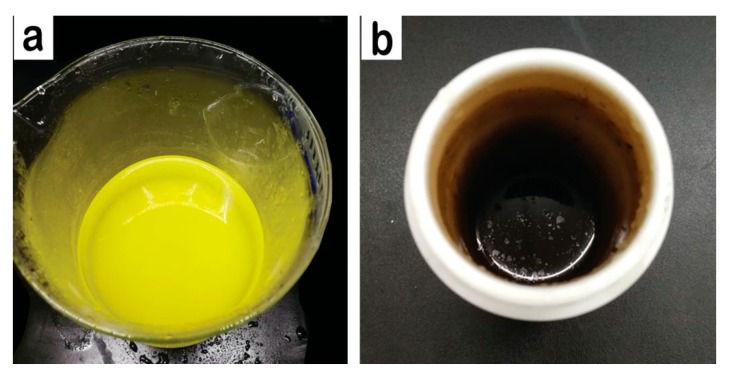
(**a**) Filtered CQDs solution; (**b**) dialysis CQDs solution.

CQDs were synthesized by a hydrothermal method. Firstly, the Radiata pine woody powder (1.3 g) was dispersed into purified water (70 mL) under a strong stirring. Then, the mixture solution was transferred into a high-pressure Teflon-lined stainless-steel autoclave (100 mL, Taizhou, China) and heated at 200 °C for 8 h in an oil bath. After being cooled down to room temperature, the resultant light-yellow product (Figure 1a) was removed out from the autoclave and then filtered through a piece of microporous membrane (pore size, 0.22 µm), to remove large particles. Brown–yellow CQDs (Figure 1b) were obtained by a dialysis treatment for 48 h, with a dialysis membrane (MWCO, 500–1000 Da, Spectrum Labs, Los Angeles, CA, USA). The resulting CQDs (with a concentration of 0.1 wt.%) were stored in a refrigerator (4 °C) for later use.

### 2.3. Fabrication of PVA-Based Films

#### 2.3.1. Experimental Method

Table 1 shows the experimental method for preparation of PVA-based films. No. 1 (PVA film) and No. 6 (PVA/CNF film) are the control to No. 2–5 and No. 7–10, respectively, for investigating the influence of CQDs on the properties of PVA films and PVA/CNF composites.

#### 2.3.2. Fabrication of PVA-Based Films

A 10 wt.% PVA solution was obtained by mixing PVA (10 g) and purified water (90 mL) at 95 °C for 2 h, with stirring, and 1 wt.% CNF solution was prepared by diluting the purchased CNF solution (8 g, 2.5 wt.%) with deionized water (12 mL), under strong stirring at room temperature for 1 h. PVA solution (10 wt.%) and CNF solution (1 wt.%) were mixed according to the formula in Table 1, and then ultrasonic-processed (XO-1200, Nanjing Xianqu Biological Technology Co., Ltd., China) in an ice/water bath for 40 min at 20–25 kHz frequency, with an output power of 960 W, resulting in PVA/CNF mixture for later use.

**Figure 2:**
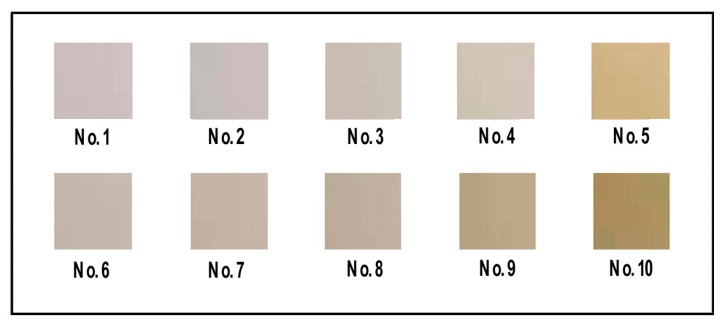
Images of PVA, PVA/CQDs, PVA/CNF, and PVA/CNF/CQDs films under daylight.

PVA film (No. 1, shown in Figure 2) was obtained by placing 10 mL of PVA solution (10 wt.%) in a petri dish and drying it at 40 °C for 24 h (101-2BS, Beijing Hengnuolixing Technology Co., Ltd., China). PVA/CNF film (No. 6, shown in Figure 2) was prepared in the same manner.

According to the formulation in Table 1, a certain amount of CQDs were mixed with PVA solution or PVA/CNF solution. After ultrasonic processing (XO-1200, Nanjing Xianqu Biological Technology Co., Ltd., China) in an ice/water bath for 30 min at 20–25 kHz frequency, with an output power of 960 W, the mixture was cast in a glass petri dish with a diameter of 90 mm and oven-dried with ventilation (101-2BS, Beijing Hengnuolixing Technology Co., Ltd., China) at 60 °C, to a state of smooth demolding, resulting in PVA/CQDs films (No. 2–5, shown in Figure 2) and PVA/CNF/CQDs films (No. 7–10, shown in Figure 2).

#### 2.3.3. Characterizations of the Obtained CQDs

A three-use ultraviolet analyzer (ZF-1, Li Chen, Zhejiang, China) with an emission wavelength of 365 and 254 nm was used to obtain the fluorescence photographs of the prepared CQDs. The morphology of the CQDs was investigated with a cold field-emission scanning electron microscope (FE-SEM, S-4700, Hitachi, Japan) with an accelerating voltage of 200 kV, and the photoluminescence (PL) spectra and photoluminescent intensity were recorded, using an LS 55 fluorescence spectrophotometer (F-7000, Hitachi, Japan) with a band pass for excitation and emission of 10.

The quantum yield (QY) of the CQDs in this study was determined by referring to the method reported in the literature [43]. Quinine sulfate was dissolved in 0.1 mL of H_2_SO_4_ (Φ = 54%) as a standard. In order to minimize inner filter effect, both absorbance of the CQDs and the quinine sulfate solutions were adjusted to below 0.1. The QY was calculated by Equation (1):

(1) $$QY_{X}=QY_{ST}(\frac{I_{X}}{I_{ST}})(\frac{A_{ST}}{A_{X}}){\left(\frac{\eta_{X}}{\eta_{ST}}\right)}^{2}$$

where *I* and *A* are the fluorescence integral intensity and absorbance, respectively; *η* the refractive index of the solvent is 1.33; and the subscripts *_X_* and *_ST_* correspond to CQDs and quinine sulfate, respectively. According to the PL spectra of the CQDs and the quinine sulfate, the QY of our CQDs is 1.60%.

#### 2.3.4. Characterizations of PVA-Based Films

The photoluminescent spectra and photoluminescent intensity of the PVA-based films were tested by using the same method as in Section 2.3.3. In addition, ZF-1 Ultraviolet Analyzer was used to study whether the prepared films had fluorescence effect and the influence of CQDs on fluorescence intensity of the films. The Fourier Transform infrared (FTIR) spectra of the PVA-based films were recorded on a spectrometer (VERTEX 80V, Bruker, Hamburg, Germany), using the KBr pellet technique, with a resolution of 2 cm^−1^ in the range of 500–4000 cm^−1^. The ultraviolet-visible absorption characteristics of the films were measured with an UV-vis spectrophotometer (Lambda 950, Perkin Elmer, Waltham, MA, USA). The PVA-based film was cut to pieces with a diameter of 30 mm, after drying in an oven (Electric blast drying box, 101-2BS, Beijing Hengnuolixing Technology Co., Ltd., Beijing, China) at 90 °C for 24 h. The surface contact angles measurement of the prepared films to distilled water was tested with an automatic single fiber contact angle measuring instrument (OCA40, Data Physics Instruments GmbH, Feldstadt, German). In this study, we used the water absorption of the film to quantify its water-resistance. The size of the sample was 20 × 20 (mm, length × width). After being oven-dried at 90 °C for 24 h, the sample was weighed (Wi) and then immersed in distilled water (50 mL) for 24 h, at room temperature. After we dried the water on the sample’s surface with napkins, the sample was weighed again (Wf). The water absorption of the sample was calculated by Equation (2):

(2) $$A=\left[\left(W_{f}-W_{i}\right)/W_{i}\right]\times 100\%$$

The tensile test was done by using a universal mechanical tester (CMT 4204, 220V, Shenzhen Sans Testing Machine Co., Ltd., Guangdong, China), at a relative humidity of 50% and a loading speed of 10 mm/min. The size of the sample was 50 × 20 (mm, length × width). Three specimens were tested for each film, and the average values were reported.

## 3. Results and Discussion

### 3.1. Optical Performance of the Prepared CQDs

**Figure 3:**
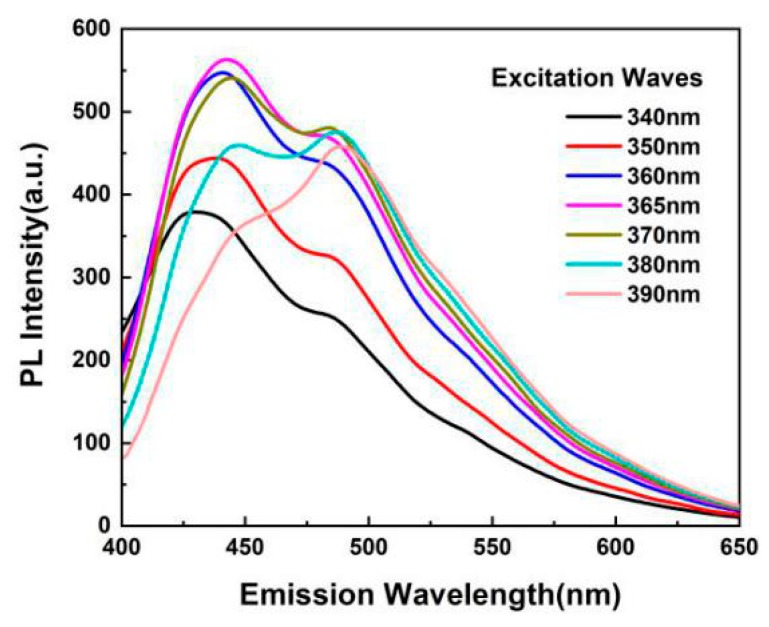
PL intensity of the diluted CQDs (0.1 wt.%) solution at various excitation wavelengths.

The Radiata-pine-derived CQDs synthesized by hydrothermal method shows good fluorescence photoluminescence, and the fluorescence intensity of the prepared CQDs was affected by excitation waves (340–390 nm), as shown in Figure 3. When the excitation wavelength was increased from 340 to 390 nm, the emission peak became slightly red-shifted, which was induced by the condensation between CQDs and PVA [44]. When the excitation wavelength of ultraviolet ray was 340–390 nm, the wavelength of the strongest emission peak of the CQDs was 400–450 nm, which belongs to blue–violet light. Moreover, when the excitation wavelength was 365 nm, the CQDs presented the strongest fluorescence intensity (563 a.u.), and a corresponding emission wavelength was 423 nm, which belongs to violet light. This indicates that, in the PL spectrum of our CQDs, there appears an excitation wavelength-dependent feature. The previous study also found similar results [43,45].

**Figure 4:**
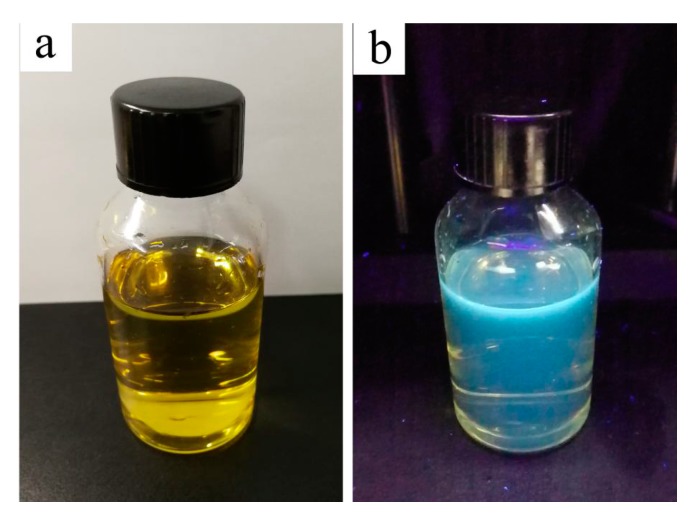
Optical images of the prepared CQDs solution under daylight (**a**) and UV light (**b**).

Figure 4 shows the optical images of the prepared CQDs under daylight (a) and UV light (c). The CQDs solution is yellowish and transparent under daylight; however, it emits violet fluorescence under UV light, with a wavelength of 365 nm. This is consistent with the finding in Figure 3. As for the mechanism of CQDs with fluorescence photoluminescence, it is not well understood [44]. It may be affected by such factors as electronic conjugate structures [46], emissive traps [47], and free zig-zag sites [48]. Cunjin Wang, et al. believed that S-CQDs reduced the non-radiative transition of electrons due to fewer surface defects, causing more electrons to radiate in the form of photons, therefore resulting in strong blue fluorescence of S-CQDs under 365 nm ultraviolet lamps [49].

### 3.2. PL Property of the Prepared PVA-Based Films

**Figure 5:**
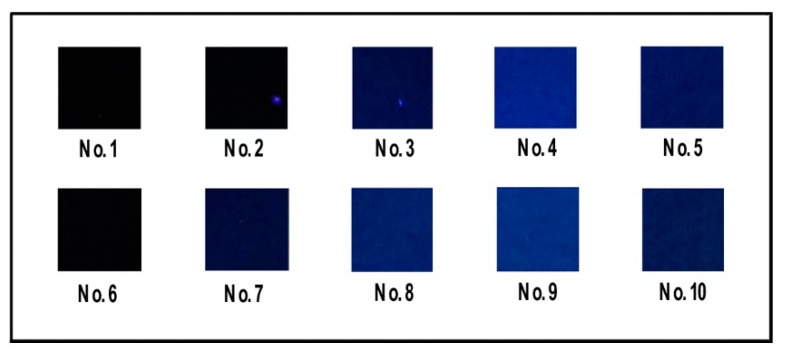
Images of PVA, PVA/CQDs, PVA/CNF, and PVA/CNF/CQDs films under ultraviolet rays (365 nm).

Figure 5 presents the images of the prepared PVA, PVA/CQDs, PVA/CNF, and PVA/CNF/CQDs films under ultraviolet rays (UV-rays) with a wavelength of 365 nm. The sample No. 1 (pure PVA film) has no fluorescence under UV-rays. When the load of CQDs in PVA/CQDs composites increases from 0.2 to 2 mL (from No. 2 to No. 4), the fluorescence intensity of the films increases sequentially; this is due to the increasing dosage of CQDs in the composites. However, further increasing the content of CQDs to 4 mL (No. 5) leads to a decrease of fluorescence intensity. This is due to the conjugation effect and the fluorescence self-quenching caused by too many CQDs in the composites [50,51]. Thus, introducing our CQDs into PVA films can endow PVA/CQDs composites with certain photoluminescence, but the content of CQDs in the composite has a threshold, which is 2 mL.

**Figure 6:**
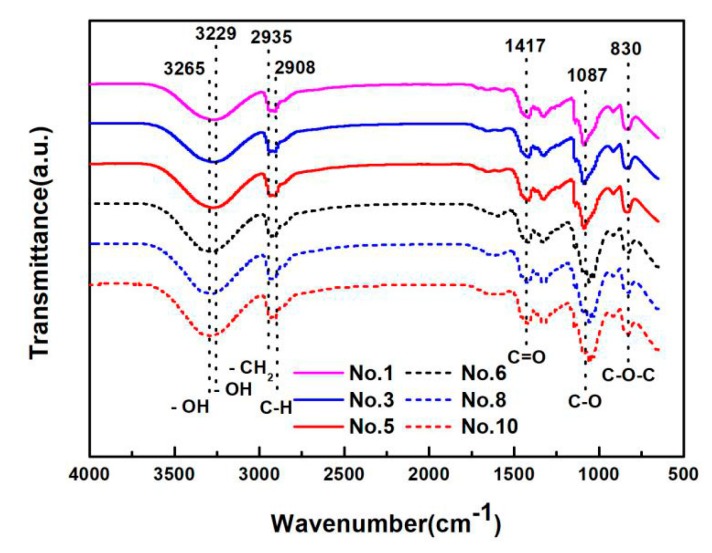
FTIR spectra of PVA, PVA/CQDs, PVA/CNF, and PVA/CNF/CQDs films.

Similarly, the introduction of our CQDs into PVA/CNF composites can endow PVA/CNF/CQDs films with certain fluorescence photogenic effect. Sample No. 6 (PVA/CNF film) has no fluorescence under UV-rays, indicating that the CNF in the PVA/CNF composites has no contribution to fluorescence photogenic effect of PVA/CNF films. The fluorescence intensity of the prepared PVA/CNF/CQDs films gradually enhances with increasing the dosage of the CQDs from 0.2 to 2 mL (from No. 7 to No. 9). When this dosage exceeds 2 mL (No. 10), the fluorescence intensity of the PVA/CNF/CQDs films no longer increases, or even starts to decrease. This trend is consistent with some previous research results; it is the effect of the Mg–N-CQDs concentration was investigated, showing that the quenching efficiency of Hg(II) was decreased with increasing concentration of Mg–N-CQDs, which confirmed by Liu et al. [52]. Thus, just like PVA/CQDs films, there is also a threshold value for the CQDs content in PVA/CNF/CQDs composites, which is 3 mL. It indicates that CNF may delay the occurrence of quenching phenomenon due to too many CQDs in the composites. In the structure of the composites, the CNF and PVA are cross-linked by hydrogen bonds in Figure 6 to generate the tension, and the CQDs surface atoms are subjected to external strain from their relaxation positions, thus generating new energy states in the CQDs band-gap. These states provide a non-radiate recombination path for quenching [53].

### 3.3. Optical Performance of the Prepared PVA-Based Films

#### 3.3.1. PL Spectra of the Prepared PVA-Based Films

**Figure 7:**
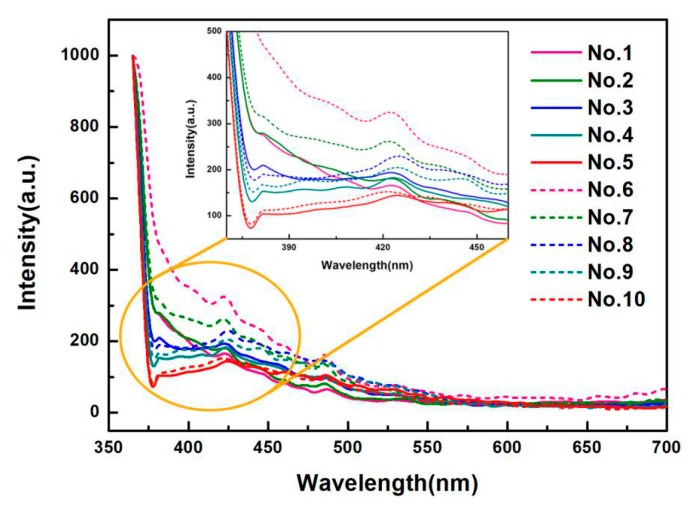
PL spectra of PVA, PVA/CQDs, PVA/CNF, and PVA/CNF/CQDs films (No. 1–10).

Figure 7 displays the optical performance of the prepared films with various contents of CQDs (No. 1 to No. 10), at the excitation wavelength of 365 nm. It is observed that the fluorescence spectra of all the films present a similar changing trend. The fluorescence intensity decreases significantly with the increase of emission wavelength from 350 to 375 nm, which belongs to the band of ultraviolet light. However, when the emission wavelength increases from 425 nm to 575 nm, the fluorescence intensity of the films decreases slowly in the visible light band, and tends to be almost the same, even quenching for the emission wavelength greater than 575 nm. Moreover, the fluorescence spectra are clearly visible with convex peaks in the emission wavelength of 375 to 450 nm, which is the lipid oxidation fluorescence peak. The reason is that 2p2 nonbonding electrons of the oxygen atom occur n → π * electron transition in the free hydroxyl group of the molecular conformation of PVA [54]. From this, we conclude that PVA-based films dopes with CNF and CQDs. It shows that the addition of CNF and CQDs does not affect the characteristic emission peak of PVA-based films; however, the fluorescence intensity is affected.

With the increase of CQDs content from 0 to 4 mL, the light intensity of emission waves with a wavelength of 375–410 nm emitted by the films gradually decreases, showing that the CQDs content has a significant effect on the attenuation of the fluorescence intensity of composites, which is consistent with the result of Ling [55]. One possible reason is due to the increase of electron-withdrawing group (carbonyl group) in the films, which can weaken the fluorescence [54]. Another possibility may be due to the π → π * and n → π * electron transitions to improve after doping CQDs in PVA, PVA/CNF films (No. 1 and No. 6), thus weakening the fluorescence intensity of the films [56].

In general, the emission intensity (with same wavelength) of the PVA/CNF/CQDs films is higher than that of the PVA/CQDs composites, indicating that introducing CNF into the PVA/CQDs films leads to an increase of emission intensity. The reason is maybe that the absorption ability of excited photons is enhanced by adding CNF, or due to existing the PVA/CNF hydrogel [57] with excellent fluorescence [58], which leads to the increase of fluorescence intensity of PVA/CNF/CQDs films [59]. In addition, the optimum emission peak of the PVA/CQDs films is in 418–421 nm, while that of PVA/CNF/CQDs films is in 422–425 nm, which creates the red-shift phenomenon. It is due to the introduction of CNF.

#### 3.3.2. FTIR Spectra of the PVA-Based Films

Figure 6 shows FTIR spectra of the PVA, PVA/CQDs, PVA/CNF, and PVA/CNF/CQDs films, and Table 2 lists the typical absorption bands in the spectra and their corresponding functional groups. The broad and intense peaks at 3265 and 3229 cm^−1^ correspond to the –OH stretching vibration [60], while the peak at 2935 cm^−1^ is attributed to the asymmetric bending vibration of –CH_2_ [61]. The absorption band at 2908 cm^−1^ is related to the C–H stretching vibration, which is similar to the result of Saikia [62]. The peaks at 1417, 1087, and 830 cm^−1^ are associated with the stretching vibration of nonconjugated C=O [63], the stretching vibration of C–O [61], and the asymmetric aromatic ring skeleton vibration of C–O–C [30], respectively. The vibration frequency of the peaks at 2908, 1417, and 1087 cm^−1^ distributes sparsely due to the electron transition, which leads to the weaker absorption bands. However, the stronger absorption bands appear at 2935 and 830 cm^−1^ due to a large variation of dipole moment caused by the asymmetric molecular vibrations. The presence of these surface-functional groups, as shown in Table 2, results in the high hydrophilic solubility of the prepared films (No. 1, No. 3, No. 5, No. 6, No. 8, and No. 10 in Table 1).

There are no significant differences among the spectra of the pure PVA, PVA/CQDs, PVA/CNF, and PVA/CNF/CQDs films (No. 1, No. 3, No5, No. 6, No. 8, and No. 10 in Table 1) in Figure 6, indicating that the introduction of CNF (0.8 wt.%) or CQDs (1 and 4 mL, as shown in Table 1) into PVA does not cause significant functional group changes in PVA films. This is due to the fact that the infrared spectra of the prepared films do not deviate greatly, because the main component of the prepared films is PVA.

Compared with the spectra of the PVA, PVA/CQDs, PVA/CNF, and PVA/CNF/CQDs films (No. 1, No. 3, No. 5, No. 6, No. 8, and No. 10 in Table 1), the absorption peaks are shifted from 3229 to 3265 cm^−1^, which may be due to the hydrogen bonds between crosslinking [64]. Compared with the spectra of the PVA and PVA/CQDs films (No. 1, No. 3, and No. 5 in Table 1), the peak at 1087 cm^−1^ in infrared spectra of the PVA/CNF and PVA/CNF/CQDs films (No. 6, No. 8, and No. 10 in Table 1) shifts lightly to right. There are two possible reasons for this shift: The one reason is the existence of π-π * conjugate effect due to the sp2 Hybrid when the C–O group in the films links to the larger groups of conjugate system; the other is that it is easily formed through the intramolecular hydrogen bonding between hydroxyl groups and carbonyl groups [65]. It shows that the absorption peaks are strongly related to the oxidation functional groups, which is that the PVA/CNF/CQDs films is basically composed of oxygen atoms with amorphous carbon bonds [60].

The results show that no new chemical bonds are produced in the PVA/CQDs, PVA/CNF, and PVA/CNF/CQDs films (No. 3, No. 5, No. 6, No. 8, and No. 10 in Table 1).

### 3.4. Barrier Property to Light of the Prepared PVA-Based Films

**Figure 8:**
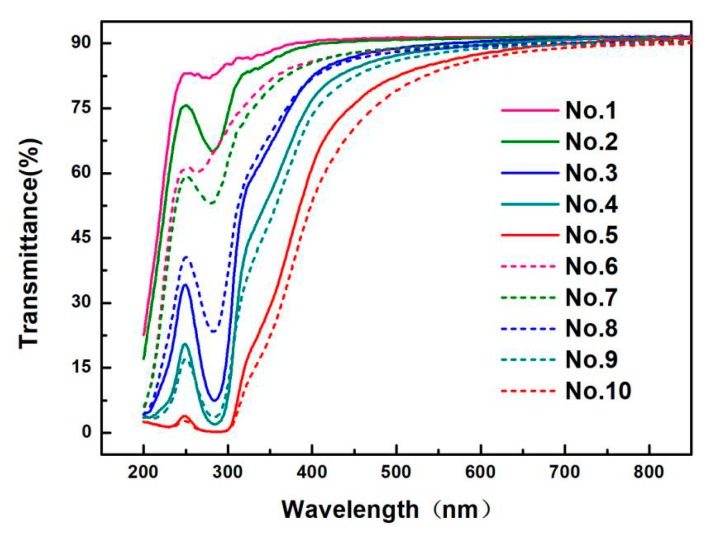
Transmittance of the PVA, PVA/CQDs, PVA/CNF, and PVA/CNF/CQDs films.

Transmittance curves of the films with different CQDs contents are shown in Figure 8. All the films (No. 1–10) show a common trend; that is, when the wavelength changes from 290 nm to 800 nm, the transmittance increases gradually until it becomes stable, reaching about 90%. It indicates that the films have worse barrier property to light after the 700 nm wavelength. However, a strong absorption peak appears at the wavelength from 240 to 290 nm, which belongs to C–H bend peak of a vibrational structure. It is due to the dipole–dipole interaction between molecules [66], or n → π * transition, because of unsaturated (C=O) bond break, as reported earlier [67].

Compared with the pure PVA films and PVA/CQDs films, the transmittance of PVA/CNF films and PVA/CNF/CQDs films decreases with the addition of CNF (8 wt.%) into PVA-based film, showing that barrier property to light of PVA/CNF film improved by the introduction of CNF. For PVA/CQDs films and PVA/CNF/CQDs films, the transmittance of the films decreased with the increase of the dosage of CQDs. It indicates that barrier property to light of the films enhances after the addition of CQDs. The result coincides with Liu [68]. It is attributed to the increase of cluster size doped CQDs [69]. The pure PVA film has almost no barrier to UV light, while the transmittance of the films in the UV-light region reduces after adding CNF or CQDs; especially around 280 nm, the transmittance is very low, which shows that the PVA films with the added CNF or CQDs have excellent barrier to UV light. Furthermore, it is observed that, when the CQDs are added up to 4 mL (No. 5 and No. 10), the transmittance of PVA/CQDs and PVA/CNF/CQDs films is almost down to zero in UV wavelength range of 200–310 nm, demonstrating that the PVA/CQDs and PVA/CNF/CQDs films with CQDs dosage of 4 mL in the composite matrix have good barrier property of UV light.

In addition, for PVA film (No. 1 in Table 1) and PVA/CQDs films (No. 2 to No. 5 in Table 1), the peaks of the light transmittance curve shift to the right at UV wavelength range of 240 to 300 nm, with the increase of CQDs contents. One reason is that, although the spectra of PVA/CQDs films (No. 2 to No. 5 in Table 1) are similar to those of the parent compound PVA, the observed shift in the band positions may be attributed to the substituent chains attached. Another reason is the mutual interactions of the CQDs surface dangling bonds, the absorption peak with CQDs added happens to the red-shift in the UV-visible absorption spectrum, which generates the self-reabsorption. This red-shift overlaps with emission, causing re-absorption with the loss of energy [70]. Compared with the PVA/CNF films (No. 6 in Table 1), the PVA/CNF/CQDs films (No. 7–10 in Table 1) shift to right; this is consistent with our previous reasons. Consequently, the advantage of CQDs to PVA films is the reduction of the UV-transmittance [66].

As a result, it is shown that the modified PVA-based film can block almost all of the UV region, so the PVA/CNF/CQDs films can be used to block UV-rays’ materials.

### 3.5. Tensile Properties of the Prepared PVA-Based Films

**Figure 9:**
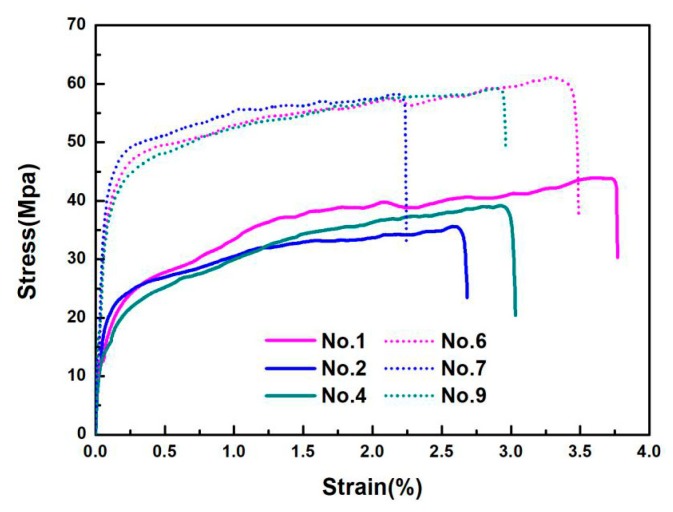
Stress–strain curves of the PVA-based films.

**Figure 10:**
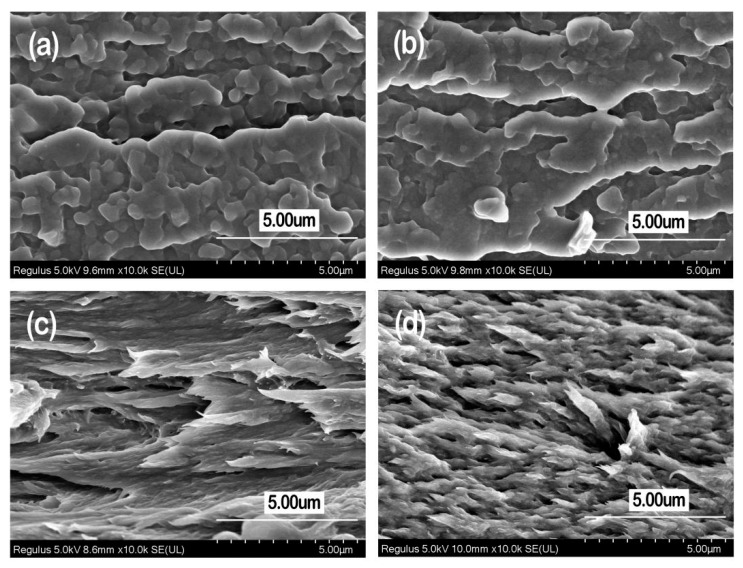
SEM graphs of the fracture section of PVA film, No. 1 (**a**); PVA/CQDs film, No. 4 (**b**); PVA/CNF, No. 6 (**c**); and PVA/CNF/CQDs film, No. 9 (**d**).

The tensile properties of the prepared PVA-based films are listed in Table 3, and the typical stress–strain curves are shown in Figure 9. Figure 10 gives SEM graphs of the fracture section of PVA, PVA/CQDs, PVA/CNF, and PVA/CNF/CQDs films. As shown in Figure 9, the preferential tensile orientation is observed in the fractured surface of all the PVA-based films, which results in the “drawing” at strains when the stress reaches a plateau before the film failure [71], as shown in Figure 9.

When adding an 8 wt.% CNF into the PVA matrix, the tensile modulus and tensile strength increases from 580.76 and 42.50 MPa (No. 1 in Table 1) to 1291.00 and 51.68 MPa (No. 6 in Table 1), respectively. It indicates that the introduction of CNF to the PVA-based film (No. 5 in Table 1) presents a higher tensile modulus and tensile strength, as well as a lower elongation at break, demonstrating that the CNF in PVA/CNF matrix can significantly improve its tensile performance. This is mainly attributed to the stable hydrogen bond between the CNF and PVA polymer matrix, which restricts propagation of the crack [72]. Compared with the smooth and flat tensile failure section of PVA film (Figure 10a), the failure section of PVA/CNF film (Figure 10c) is rough and uneven, which is evidence of a good combination of PVA and CNF interface. Yan et al. also found that the CNF could beneficially improve the ductility and tensile strength of PVA/CNF films [73].

As shown in Table 3 and Figure 9, introducing CQDs into PVA or PVA/CNF matrix leads to an increase of tensile modulus and tensile strength of the composite. The tensile modulus and tensile strength of the PVA/CQDs films with 0.2 mL CQDs (No. 2) were 42.03% and 14.3% higher than PVA (No. 1), respectively; and when the dosage of CQDs increases to 2 mL in the matrix, the tensile modulus and tensile strength of the PVA/CQDs film (No. 4) increases by 51.75% and 8.02%, respectively. Similarly, the tensile modulus and tensile strength of the PVA/CNF/CQDs film with 2 mL CQDs (No. 9) was 1.90% and 15.60% higher than the PVA/CNF film (No. 6), respectively. Although we cannot explain for the time being why CQDs can enhance the tensile modulus and tensile strength of PVA or PVA/CNF films, we hypothesize it may be the result of hydrogen bonds, van der Waals force bond, and other chemical bonds between the active groups on the surface of CQDs and PVA and CNF. The SEM graphs in Figure 10 support this hypothesis. Compared with the PVA/CNF film (Figure 10c), the PVA/CNF/CQDs film (Figure 10d) presents a denser failure section, which is evidence of a better combination of PVA, CNF, and CQDs interface.

As described in Section 3.4, the introduction of CQDs into PVA or PVA/CNF gives PVA-based composites a certain UV-light-barrier property; and the tensile test results here show that the CQDs in PVA or PVA/CNF matrix improve the tensile strength and modulus of the composite films. Compared with the proper films used in packaging [74], the prepared PVA/CQDs and PVA/CNF/CQDs films in this research study have higher mechanical properties and UV-radiation protection. Therefore, the prepared films have promising applications in the packaging field.

### 3.6. Water-Resistance of the Prepared PVA-Based Films

**Figure 11:**
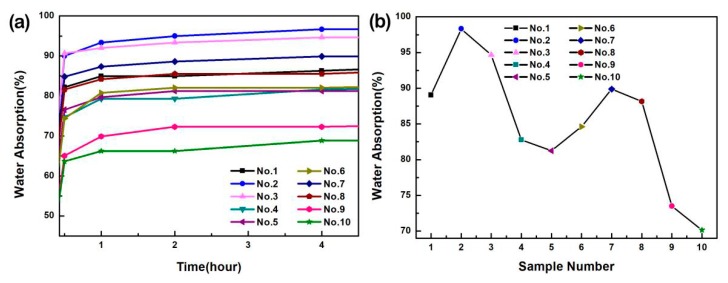
(**a**) Water absorption of the PVA, PVA/CQDs, PVA/CNF, and PVA/CNF/CQDs films with different contents of CQDs in 4 h. (**b**) Average water absorption of the PVA, PVA/CQDs, PVA/CNF, and PVA/CNF/CQDs films with different contents of CQDs within 24 h.

Water-barrier property of the prepared PVA-based films is shown in Figure 11a. The water absorption of films changes significantly within 0.5 h, which is due to the hydrophilic character of CNF and CQDs [75,76]. In addition, the average value of the water absorption of the PVA film (No. 1), PVA/CQDs film (No. 2 to No. 5), PVA/CNF film (No. 6), and PVA/CNF/CQDs film (No. 7 to No. 10) was 82.19%, 82.99%, 74.36%, and 77.15% after 0.5 h, reaching 89.04%, 89.25%, 84.62%, and 84.70% after 24 h, respectively. It shows that the water absorption of films is slightly unchanged after 0.5 h, which is due to saturation.

It shows the trend of the water absorption of the 10 composites is presented after 24 h in Figure 11b. The water absorption is a vital index to characterize the water-resistance of composites. With the decrease of water absorption, the water-resistance of the material increases. Compared with the PVA-based films, the water absorption of PVA/CQDs films (0.2 mL) is higher, which maybe be attributed to the introduction of CQDs. Meanwhile, the water absorption of PVA/CNF/CQDs films (0.2 mL) is higher than that of PVA/CNF films, which is consistent with the previous one; that is, the water absorption increase of the films is due to the introduction of CQDs. In addition, when the content of CQDs exceeds 0.2 mL, the water absorption of PVA/CQDs (No. 3 to No. 5) and PVA/CNF/CQDs (No. 8 to No. 10) films decreased significantly, which may be due to the reduction of porosity [77]. Another reason is, when CQDs with many carboxyl groups are introduced, the decrease of free hydroxyl radicals is due to the reaction between hydroxyl and carboxyl groups. This leads to the decrease of water absorption of the films [78]. This indicates that the change of CQDs content could affect the water absorption of the films. The water absorption decreases from 89% of the pure PVA films to 81.25% of the PVA/CQDs films (No. 5), and reduces to 70.13% of the PVA/CNF/CQDs films (No. 10). Compared with the water absorption of PVA/CQDs (No. 1–No. 5), the water absorption of the PVA/CNF/CQDs (No. 6 to No. 10) films reduces significantly. The result shows that the introduction of CNF reduces the water absorption of PVA/CQDs-based films [79]. When doping CNF in the PVA/CQDs films, the composites form more hydrogen bonds to reduce the amount of hydrophilic hydroxyl. Therefore, the surface polar groups of the PVA-based films have less contact with water molecules [80]. The results show that the doping of CNF and CQDs improves the water-resistance of PVA-based films.

### 3.7. Surface Wettability of PVA-Based Films

**Figure 12:**
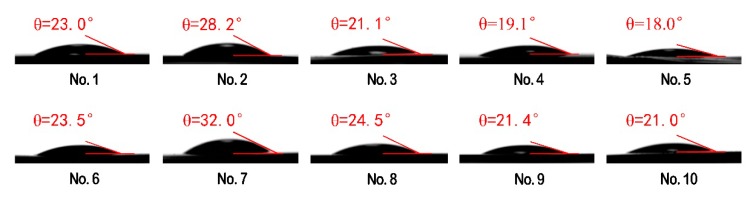
Water contact angles of the PVA/CQDs films and the PVA/CNF/CQDs films with different contents of CQDs.

The contact angles of the 10 PVA-based composites are presented in Figure 12. The water contact angle is an important index to evaluate surface wettability of the prepared films. The contact angles of the films (No. 1 to No. 10) fall within a narrow range (18°–32°), due to the fact that the composites have a large amount of the hydrophilic groups. In contrast, the pure PVA films (No. 1) and the PVA/CNF films (No. 6) have contact angles of 23°and 23.5°, respectively, which are lower than those of the PVA-based films (28.2°) and PVA/CNF-based films (32.0°) loaded with 0.2 mL CQDs. This suggests that the wettability of PVA/CQDs films (No. 2) and PVA/CNF/CQDs films (No. 7) is worse than that of the pure PVA films and the PVA/CNF films, respectively. The change of contact angle is mainly related to the surface smoothness, porosity, pore size, and distribution of the films. This is probably due to the slight increase of surface smoothness of the prepared films after addition of CQDs (<0.2 mL), which is shown in Figure 10 [81], and is in good agreement with existing work [82]. Compared with the pure PVA films, the PVA/CQDs films (No. 3 to No. 5) show lower contact angles, which is attributed to the large amount of hydroxyl formed by high content of CQDs in the films. This happens for the same reason that the PVA/CNF/CQDs films (No. 8 to No. 10) have lower contact angles. Furthermore, the contact angles of PVA/CQDs films (No. 3 to No. 5) and PVA/CNF/CQDs films (No. 8 to No. 10) are small, which is due to no new chemical functional groups generated. This indicates that the hydrophilicity of the films is not changed with the introduction of CQDs. With the increase of CQDs content, the water contact angle decreases, which leads to the wettability of the prepared films improves, due to a change in surface properties of the blend films. Compared with the PVA/CQDs films (No. 1 to No. 5), respectively, the PVA/CNF/CQDs films (No. 6 to No. 10) present lightly larger water contact angle (<32°), due to the slightly higher surface roughness of the films (No. 6 to No. 10) after the introduction of CNF [83], which can be observed from the SEM graphs of the PVA/CNF/CQDs films in Figure 10. The results show the films after the introduction of CNF show a worse wettability.

## 4. Conclusions

In summary, a one-step hydrothermal method was adopted to prepare CQDs from Radiata pine at a constant temperature of 200 °C for 8 h for the first time. The synthesized Radiata-pine-derived CQDs exhibited excitation-dependent PL emissions with a QY of 1.60%.

When the excitation wavelength was 365 nm, the CQDs presented the strongest blue fluorescence intensity (563 a.u.). Introducing our CQDs into PVA films can endow PVA-based composites with certain photoluminescence; the fluorescence of films increases at first and then descends, reaching self-quenching with the increase of CQDs dosage in the composites. Meanwhile, CNF contributes to the fluorescence of the composite film, which creates the red-shift phenomenon at optimum emission peak. In addition, doping CNF and CQDs in the PVA-blend films is also confirmed by FT-IR, which indicates that the PVA matrix’s introduction to CNF and CQDs went well, due to no new chemical bonds being produced. The pure PVA film has almost no barrier to UV light, while the transmittance of the films in the UV-light region reduces after adding CNF or CQDs; especially around 280 nm, the transmittance is very low, which shows that the PVA films adding CNF or CQDs have excellent barrier to UV light.

Dramatically, introducing CQDs and CNF into PVA matrix leads to an increase of tensile modulus and tensile strength of the composite. Demonstrating the CNF in PVA/CNF matrix can significantly improve its tensile performance. The introduction of CQDs affects the water absorption of PVA/CQDs based films. When doping CNF in the PVA/CQDs films, the composites form more hydrogen bonds to reduce the amount of hydrophilic hydroxyl. Therefore, the doping of CNF and CQDs improves the water-resistance of PVA-based films. A significant reduction in the contact angle of the PVA-modified film is observed, suggesting that the functionalized PVA improved the wettability of the film’s surface. With the increase of CQDs content, the water contact angle decreases, which leads to an improvement in the wettability of the prepared films.

Overall, the improvement in water-resistance property, enhancement of the physical properties (structural and mechanical), and UV barrier of these the modified PVA-based films makes a variety of uses in the multifunctional applications. For example, the composites have broad prospects in the application of fruit and vegetable packaging. Therefore, we believe that the composite films can be used to make transparent, anti-counterfeiting, anti-UV, good-mechanical-strength materials, which have better application prospects in future.

## Figures and Tables

**Table 1:** Experimental method for preparation of PVA-based films.

Film No.	CQDs (0.1 wt.%)/mL	PVA (10%)/mL	CNF (1%)/mL
No. 1	0	10	0
No. 2	0.2	10	0
No. 3	1	10	0
No. 4	2	10	0
No. 5	4	10	0
No. 6	0	10	10
No. 7	0.2	10	10
No. 8	1	10	10
No. 9	2	10	10
No. 10	4	10	10

**Table 2:** Functional group absorption bands of the PVA, PVA/CQDs, PVA/CNF, and PVA/CNF/CQDs films.

Wavenumber (cm^−1^)	Functional Groups	Vibrations
3265, 3229	–OH	stretching
2935	–CH_2_	bending
2908	C–H	stretching
1417	C=O	stretching
1087	C–O	stretching
830	C–O–C	asymmetric aromatic ring skeleton

**Table 3:** Tensile properties of the prepared PVA-based films.

Film Number	Tensile Modulus (MPa)	Tensile Strength (MPa)	Elongation at Break (%)	Film Thickness (mm)
No. 1	580.76 (98.27 ^a^)	42.50 (2.42 ^a^)	345.02 (25.49 ^a^)	0.129 ^b^
No. 2	824.84 (121.76 ^a^)	48.58 (6.68 ^a^)	302.83 (48.24 ^a^)	0.115 ^b^
No. 4	881.28 (125.59 ^a^)	45.91 (3.42 ^a^)	287.69 (32.89 ^a^)	0.177 ^b^
No. 6	1291.00 (245.55 ^a^)	51.68 (6.51 ^a^)	254.77 (54.01 ^a^)	0.137 ^b^
No. 7	1185.25 (121.26 ^a^)	56.37 (4.64 ^a^)	264.39 (27.02 ^a^)	0.157 ^b^
No. 9	1316.06 (164.72 ^a^)	59.71 (5.22 ^a^)	313.01 (10.43 ^a^)	0.163 ^b^

^a^ The standard deviation value of three samples. ^b^ The average value of three samples.

## References

[1] Zhu Y.K., Chen D.J. (2018). Clay-based nanofibrous membranes reinforced by multi-walled carbon nanotubes. Ceram. Int..

[2] Wu M.C., Chan S.H., Lin T.H. (2015). Fabrication and photocatalytic performance of electrospun PVA/silk/TiO2 nanocomposite textile. Funct. Mater. Lett..

[3] Hassannejad H., Nouri A., Soltani S., Molavi F.K. (2019). Study of corrosion behavior of the biodegradable chitosan-polyvinyl alcohol coatings on AA8011 aluminum alloy. Mater. Res. Express.

[4] Zhu C., Xiang Q., Liu X.Y., Dong L.M. Study on antiseptic property of water soluble polyvinyl alcohol building adhesive. Proceedings of the 2016 International Conference on Advances in Energy, Environment and Chemical Science (AEECS 2016).

[5] Abdullah Z.W., Dong Y., Davies I.J., Barbhuiya S. (2017). PVA, PVA blends, and their nanocomposites for biodegradable packaging application. Polym. Plast. Technol..

[6] Gaaz T.S., Sulong A.B., Akhtar M.N., Kadhum A.A.H., Mohamad A.B., Al-Amiery A.A. (2015). Properties and applications of polyvinyl Alcohol, halloysite nanotubes and their nanocomposites. Molecules.

[7] Arain M.F., Wang M.X., Chen J.Y., Zhang H.P. (2019). Study on PVA fiber surface modification for strain-hardening cementitious composites (PVA-SHCC). Constr. Build. Mater..

[8] Lelifajri, Nawi M.A., Sabar S., Supriatno, Nawawi W.I. (2018). Preparation of immobilized activated carbon-polyvinyl alcohol composite for the adsorptive removal of 2,4-dichlorophenoxyacetic acid. J. Water Process Eng..

[9] Baker M.I., Walsh S.P., Schwartz Z., Boyan B.D. (2012). A review of polyvinyl alcohol and its uses in cartilage and orthopedic applications. J. Biomed. Mater. Res. Part B.

[10] Kim S.J., Park S.J., Kim S.I. (2003). Swelling behavior of interpenetrating polymer network hydrogels composed of poly (vinyl alcohol) and chitosan. React. Funct. Polym..

[11] Heuschmid F.F., Schneider S., Schuster P., Lauer B., van Ravenzwaay B. (2013). Polyethylene glycol-g-polyvinyl alcohol grafted copolymer: Reproductive toxicity study in wistar rats. Food. Chem. Toxicol..

[12] Limpan N., Prodpran T., Benjakul S., Prasarpran S. (2012). Influences of degree of hydrolysis and molecular weight of poly(vinyl alcohol) (PVA) on properties of fish myofibrillar protein/PVA blend films. Food Hydrocoll..

[13] Chen C.W., Xie J., Yang F.X., Zhang H.L., Xu Z.W., Liu J.L., Chen Y.J. (2018). Development of moisture-absorbing and antioxidant active packaging film based on poly (vinyl alcohol) incorporated with green tea extract and its effect on the quality of dried eel. J. Food Process. Preserv..

[14] Hong H.Q., Liao H.Y., Chen S.J., Zhang H.Y. (2014). Facile method to prepare self-healable PVA hydrogels with high water stability. Mater. Lett..

[15] Toyoda N., Yamamoto T. (2018). Dispersion of carbon nanofibers modified with polymer colloids to enhance mechanical properties of PVA nanocomposite film. Colloid Surf. A.

[16] Yu Z., Li B.Q., Chu J.Y., Zhang P.F. (2018). Silica in situ enhanced PVA/chitosan biodegradable films for food packages. Carbohydr. Polym..

[17] Lan W.J., Zhang R., Ahmed S., Qin W., Liu Y.W. (2019). Effects of various antimicrobial polyvinyl alcohol/tea polyphenol composite films on the shelf life of packaged strawberries. LWT Food. Sci. Technol..

[18] Rowe A.A., Tajvidi M., Gardner D.J. (2016). Thermal stability of cellulose nanomaterials and their composites with polyvinyl alcohol (PVA). J. Therm. Anal. Calorim..

[19] Han J.Q., Yue Y.Y., Wu Q.L., Huang C.B., Pan H., Zhan X.X., Mei C.T., Xu X.W. (2017). Effects of nanocellulose on the structure and properties of poly (vinyl alcohol)-borax hybrid foams. Cellulose.

[20] Dufresne A. (2013). Nanocellulose: A new ageless bionanomaterial. Mater. Today.

[21] Klemm D., Kramer F., Moritz S., Lindstrom T., Ankerfors M., Gray D., Dorris A. (2011). Nanocelluloses: A new family of nature-based materials. Angew. Chem. Int. Ed..

[22] Wang H.Y., Chen C.C., Fang L., Li S.Y., Chen N., Pang J.W., Li D.G. (2018). Effect of delignification technique on the ease of fibrillation of cellulose II nanofibers from wood. Cellulose.

[23] Phanthong P., Reubroycharoen P., Hao X.G., Xu G.W., Abudula A., Guan G.Q. (2018). Nanocellulose: Extraction and application. Carbon Resour. Convers..

[24] Wang H.Y., Wu T.T., Wang X.X., Cheng X.D., Chen N., Li D.G. (2019). Effect of ethylenediamine treatment on cellulose nanofibers and the formation of high-strength hydrogels. Bioresources.

[25] Chen C.C., Li D.G., Abe K., Yano H. (2018). Formation of high strength double-network gels from cellulose nanofiber/polyacrylamide via NaOH gelation treatment. Cellulose.

[26] Ching Y.C., Rahman A., Ching K.Y., Sukiman N.L., Cheng H.C. (2015). Preparation and characterization of polyvinyl alcohol-based composite reinforced with nanocellulose and nanosilica. Bioresources.

[27] Hietala M., Sain S., Oksman K. (2017). Highly redispersible sugar beet nanofibrils as reinforcement in bionanocomposites. Cellulose.

[28] Acharya A. (2013). Luminescent magnetic quantum dots for in vitro/in vivo imaging and applications in therapeutics. J. Nanosci. Nanotechnol..

[29] Shi Y.X., Liu X., Wang M., Huang J.B., Jiang X.Q., Pang J.H., Xu F., Zhang X.M. (2019). Synthesis of N-doped carbon quantum dots from bio-waste lignin for selective irons detection and cellular imaging. Int. J. Biol. Macromol..

[30] Lei C.W., Hsieh M.L., Liu W.R. (2019). A facile approach to synthesize carbon quantum dots with pH-dependent properties. Dyes Pigments.

[31] Zhao J.X., Liu C., Li Y.C., Liang J.Y., Liu J.Y., Qian T.H., Ding J.J., Cao Y.C. (2017). Preparation of carbon quantum dots based high photostability luminescent membranes. Luminescence.

[32] Zhang L.G., Wang Y., Liu W., Ni Y.H., Hou Q.X. (2019). Corncob residues as carbon quantum dots sources and their application in detection of metal ions. Ind. Crops Prod..

[33] Yan J.Y., Hou S.L., Yu Y.Z., Qiao Y., Xiao T.Q., Mei Y., Zhang Z.J., Wang B., Huang C.C., Liu C.H. (2018). The effect of surface charge on the cytotoxicity and uptake of carbon quantum dots in human umbilical cord derived mesenchymal stem cells. Colloid Surf. B.

[34] Atchudan R., Edison T.N.J.I., Aseer K.R., Perumal S., Karthik N., Lee Y.R. (2018). Highly fluorescent nitrogen-doped carbon dots derived from Phyllanthus acidus utilized as a fluorescent probe for label-free selective detection of Fe3+ ions, live cell imaging and fluorescent ink. Biosens. Bioelectron..

[35] Chandra S., Singh V.K., Yadav P.K., Bano D., Kumar V., Pandey V.K., Talat M., Hasan S.H. (2018). Mustard seeds derived fluorescent carbon quantum dots and their peroxidase-like activity for colorimetric detection of H_2_O_2_ and ascorbic acid in a real sample. Anal. Chim. Acta.

[36] Arul V., Sethuraman M.G. (2018). Facile green synthesis of fluorescent N-doped carbon dots from Actinidia deliciosa and their catalytic activity and cytotoxicity applications. Opt. Mater..

[37] Atchudan R., Edison T.N.J.I., Lee Y.R. (2016). Nitrogen-doped carbon dots originating from unripe peach for fluorescent bioimaging and electrocatalytic oxygen reduction reaction. J. Colloid Interface Sci..

[38] Wang X., Yang P., Feng Q., Meng T.T., Wei J., Xu C.Y., Han J.Q. (2019). Green Preparation of Fluorescent Carbon Quantum Dots from Cyanobacteria for Biological Imaging. Polymers.

[39] Dong L., Xiong Z.R., Liu X.D., Sheng D.K., Zhou Y., Yang Y.M. (2019). Synthesis of carbon quantum dots to fabricate ultraviolet-shielding poly(vinylidene fluoride) films. J. Appl. Polym. Sci..

[40] El-Shamy A.G. (2019). New free-standing and flexible PVA/Carbon quantum dots (CQDs) nanocomposite films with promising power factor and thermoelectric power applications. Mater. Sci. Semicond. Proc..

[41] Vandarkuzhali S.A.A., Jeyalakshmi V., Sivaraman G., Singaravadivel S., Krishnamurthy K.R., Viswanathan B. (2017). Highly fluorescent carbon dots from Pseudo-stem of banana plant: Applications as nanosensor and bio-imaging agents. Sens. Actuator B Chem..

[42] Bao R.Q., Chen Z.Y., Zhao Z.W., Sun X., Zhang J.Y., Hou L.R., Yuan C.Z. (2018). Green and facile synthesis of nitrogen and phosphorus co-doped carbon quantum dots towards fluorescent ink and sensing applications. J. Nanomater..

[43] Xue B.L., Yang Y., Sun Y.C., Fan J.S., Li X.P., Zhang Z. (2019). Photoluminescent lignin hybridized carbon quantum dots composites for bioimaging applications. Int. J. Biol. Macromol..

[44] Temerov F., Belyaev A., Ankudze B., Pakkanen T.T. (2019). Preparation and photoluminescence properties of graphene quantum dots by decomposition of graphene-encapsulated metal nanoparticles derived from Kraft lignin and transition metal salts. J. Lumin..

[45] Zhou L.F., Qiao M., Zhang L., Sun L., Zhang Y., Liu W.W. (2019). Green and efficient synthesis of carbon quantum dots and their luminescent properties. J. Lumin..

[46] Eda G., Lin Y.Y., Mattevi C., Yamaguchi H., Chen H.A., Chen I.S., Chen C.W., Chhowalla M. (2010). Blue photoluminescence from chemically derived graphene oxide. Adv. Mater..

[47] Sun Y.P., Zhou B., Lin Y., Wang W., Fernando K.A.S., Pathak P., Meziani M.J., Harruff B.A., Wang X., Wang H.F. (2006). Quantum-sized carbon dots for bright and colorful photoluminescence. J. Am. Chem. Soc..

[48] Pan D.Y., Zhang J.C., Li Z., Wu M.H. (2010). Hydrothermal route for cutting graphene sheets into blue-luminescent graphene quantum dots. Adv. Mater..

[49] Wang C.J., Wang Y.B., Shi H.X., Yan Y.J., Liu E.Z., Hu X.Y., Fan J. (2019). A strong blue fluorescent nanoprobe for highly sensitive and selective detection of mercury (II) based on sulfur doped carbon quantum dots. Mater. Chem. Phys..

[50] Hoang Q.B., Mai V.T., Nguyen D.K., Truong D.Q., Mai X.D. (2019). Crosslinking induced photoluminescence quenching in polyvinyl alcohol-carbon quantum dot composite. Mater. Today Chem..

[51] Dulkeith E., Morteani A.C., Niedereichholz T., Klar T.A., Feldmann J., Levi S.A., van Veggel F.C.J.M., Reinhoudt D.N., Moller M., Gittins D.I. (2002). Fluorescence quenching of dye molecules near gold nanoparticles: Radiative and nonradiative effects. Phys. Rev. Lett..

[52] Liu T., Li N., Dong J.X., Luo H.Q., Li N.B. (2016). Fluorescence detection of mercury ions and cysteine based on magnesium and nitrogen co-doped carbon quantum dots and IMPLICATION logic gate operation. Sens. Actuators B Chem..

[53] Zhao Q.L., Zhang Z.L., Huang B.H., Peng J., Zhang M., Pang D.W. (2008). Facile preparation of low cytotoxicity fluorescent carbon nanocrystals by electrooxidation of graphite. Chem. Commun..

[54] Htun M.T. (2012). Characterization of high-density polyethylene using laser-induced fluorescence (LIF). J. Polym. Res..

[55] Yang L., Huang B.Q., Wei X.F., Zhang W., Wang D.D. The Research on Fluorescence Intensity Attenuation of UV Fluorescent Inkjet Ink. Proceedings of the 29th International Conference on Digital Printing Technologies (NIP29)/Digital Fabrication 2013.

[56] Wang Q.Q., Zhu J.Y., Gleisner R., Kuster T.A., Baxa U., McNeil S.E. (2014). Morphological development of cellulose fibrils of a bleached eucalyptus pulp by mechanical fibrillation. Cellulose.

[57] Jing X., Li H., Mi H.Y., Liu Y.J., Feng P.Y., Tan Y.M., Turng L.S. (2019). Highly transparent, stretchable, and rapid self-healing polyvinyl alcohol/cellulose nanofibril hydrogel sensors for sensitive pressure sensing and human motion detection. Sens. Actuators B Chem..

[58] Wang Y.Q., Xue Y.A., Wang J.H., Zhu Y.P., Wang X., Zhang X.H., Zhu Y., Liao J.W., Li X.N., Wu X.G. (2019). Biocompatible and photoluminescent carbon dots/hydroxyapatite/PVA dual-network composite hydrogel scaffold and their properties. J. Polym. Res..

[59] Yan Z.D., Sun L.D., Hu C.G., Hu X.T., Zeppenfeld P. (2012). Factors influencing the ability of fluorescence emission and fluorescence quenching experimental research. Spectrosc. Spect. Anal..

[60] Ma X.T., Li S.R., Hessel V., Lin L.L., Meskers S., Gallucci F. (2019). Synthesis of luminescent carbon quantum dots by microplasma process. Chem. Eng. Process. Process Intensif..

[61] Mahmud H.N.M.E., Kassim A., Zainal Z., Yunus W.M.M. (2006). Fourier transform infrared study of polypyrrole-poly (vinyl alcohol) conducting polymer composite films: Evidence of film formation and characterization. J. Appl. Polym. Sci..

[62] Saikia M., Hower J.C., Das T., Dutta T., Saikia B.K. (2019). Feasibility study of preparation of carbon quantum dots from Pennsylvania anthracite and Kentucky bituminous coals. Fuel.

[63] Li D.P., Wu Z.J., Hang C.H., Chen L.J., Zhang Y.L., Ne Y.X. (2012). Analysis of the Character of Film Decomposition of Methyl Methacrylate (MMA) Coated Urea by Infrared Spectrum. Spectrosc. Spect. Anal..

[64] Behera B., Das P.K. (2018). Blue- and Red-Shifting Hydrogen Bonding: A Gas Phase FTIR and Ab Initio Study of RR’ CO center dot center dot center dot DCCI3 and RR’ S center dot center dot center dot DCCI3 Complexes. J. Phys. Chem. A.

[65] El-Shamy A.G. (2019). Novel conducting PVA/Carbon quantum dots (CQDs) nanocomposite for high anti- electromagnetic wave performance. J. Alloy Compd..

[66] Hertmanowski R., Biadasz A., Martynski T., Bauman D. (2003). Optical spectroscopy study of some 3,4,9,10-tetra-(n-alkoxy-carbonyl)-perylenes in Langmuir-Blodgett films. J. Mol. Struct..

[67] Baraker B.M., Lobo B. (2017). Spectroscopic Analysis of CdCl2 doped PVA-PVP Blend Films. Can. J. Phys..

[68] Lee J.H., Ko K.H., Park B.O. (2003). Electrical and optical properties of ZnO transparent conducting films by the sol–gel method. J. Cryst. Growth.

[69] Baraker B.M., Lobo B. (2018). UV irradiation induced microstructural changes in CdCl2 doped PVA–PVP blend. J. Mater. Sci. Mater. Electron..

[70] Riaz R., Ali M., Maiyalagan T., Anjum A.S., Lee S., Ko M.J., Jeong S.H. (2019). Dye-sensitized solar cell (DSSC) coated with energy down shift layer of nitrogen-doped carbon quantum dots (N-CQDs) for enhanced current density and stability. Appl. Surf. Sci..

[71] Liu D.G., Sun X., Tian H.F., Maiti S., Ma Z.S. (2013). Effects of cellulose nanofibrils on the structure and properties on PVA nanocomposites. Cellulose.

[72] Yang X.M., Shang S.M., Li L.A. (2011). Layer-structured poly (vinyl alcohol)/graphene oxide nanocomposites with improved thermal and mechanical properties. J. Appl. Polym. Sci..

[73] Wu Y., Tang Q.W., Yang F., Xu L., Wang X.H., Zhang J.L. (2019). Mechanical and thermal properties of rice straw cellulose nanofibrils-enhanced polyvinyl alcohol films using freezing-and-thawing cycle method. Cellulose.

[74] Kumar S.V., George J., Sajeevkumar V.A. (2018). PVA Based Ternary Nanocomposites with Enhanced Properties Prepared by Using a Combination of Rice Starch Nanocrystals and Silver Nanoparticles. J. Polym. Environ..

[75] Sehaqui H., Zimmermann T., Tingaut P. (2014). Hydrophobic cellulose nanopaper through a mild esterification procedure. Cellulose.

[76] Xu M.H., Zhang W., Yang Z., Yu F., Ma Y.J., Hu N.T., He D.N., Liang Q., Su Y.J., Zhang Y.F. (2015). One-pot liquid-phase exfoliation from graphite to graphene with carbon quantum dots. Nanoscale.

[77] Yin J., Deng B.L. (2015). Polymer-matrix nanocomposite membranes for water treatment. J. Membr. Sci..

[78] Oza G., Ravichandran M., Merupo V.I., Shinde S., Mewada A., Ramirez J.T., Velumani S., Sharon M., Sharon M. (2016). Camphor-mediated synthesis of carbon nanoparticles, graphitic shell encapsulated carbon nanocubes and carbon dots for bioimaging. Sci. Rep. U. K..

[79] Wang Z., Zhao S.J., Zhang W., Qi C.S., Zhang S.F., Li J.Z. (2019). Bio-inspired cellulose nanofiber-reinforced soy protein resin adhesives with dopamine-induced codeposition of “water-resistant” interphases. Appl. Surf. Sci..

[80] Shahbazi M., Rajabzadeh G., Rafe A., Ettelaie R., Ahmadi S.J. (2017). Physico-mechanical and structural characteristics of blend film of poly (vinyl alcohol) with biodegradable polymers as affected by disorderto-order conformational transition. Food Hydrocoll..

[81] Gai W.X., Zhao D.L., Chung T.S. (2019). Thin film nanocomposite hollow fiber membranes comprising Na+-functionalized carbon quantum dots for brackish water desalination. Water Res..

[82] Tang C.Y.Y., Kwon Y.N., Leckie J.O. (2009). Effect of membrane chemistry and coating layer on physiochemical properties of thin film composite polyamide RO and NF membranes I. FTIR and XPS characterization of polyamide and coating layer chemistry. Desalination.

[83] Priezjev N.V., Darhuber A.A., Troian S.M. (2005). Slip behavior in liquid films on surfaces of patterned wettability: Comparison between continuum and molecular dynamics simulations. Funct. Mater. Lett..

